# Supplementary material for: Human- or object-like? Cognitive anthropomorphism of humanoid robots
Source: PLoS One. 2022 Jul 26;17(7):e0270787. doi: 10.1371/journal.pone.0270787 (PMC9321781; doi:10.1371/journal.pone.0270787)
Supplement: S1 File — (DOCX) [file pone.0270787.s001.docx]

**Analysis of the stimuli asymmetry (Experiment 1 and 2)**

We adopted the procedure by Schmidt and Kistemarker (2015) to calculate the degree of asymmetry across the crucial stimuli employed in Experiment 1 (humans, robots and mannequins) and 2 (humans, robots with high and low levels of human-likeness). In doing so, we considered the angles of five body-axes (i.e., shoulders, elbows, hands, hips, and eyes). Unlike Schhmidt and Kistemarker (2015), we did not consider the eyes angle because some of the stimuli did not have eyes (i.e., mannequins, and robots with an overall low human-likeness). Therefore, in both experiments, we assessed every picture of robot, human, and mannequin on the four axes and calculated a mean score for each stimulus by averaging the score of each ax. A one-way ANOVA was then conducted on the asymmetry-index of the stimuli of both experiments. In Experiment 1, results showed a no significant effect of the stimulus category, *F* (2, 37.3) = 0.001, *p =* .99. Further, pairwise comparisons revealed no significant different across the three categories, *p >* .90. Similarly, in Experiment 2 the main effect of the stimuli category was not significant, *F* (2, 37.9) = 0.983, *p* = .384, and no significant differences emerged between stimulus categories, *p >* .37.

Tables A (Experiment 1) and B (Experiment 2) illustrate separate axes mean values and the stimulus category asymmetry index.

Table A. Asymmetry mean values, Experiment 1

| **Axes** |  | **Humans** | **Robots** | **Mannequins** |
| --- | --- | --- | --- | --- |
| Shoulders |  | 8.33 | 4.77 | 4.98 |
| Elbows |  | 7.62 | 8.99 | 5.96 |
| Hands |  | 12.56 | 14.68 | 14.42 |
| Hips |  | 4.08 | 4.41 | 7.29 |
| Asymmetry Index |  | **8.15** | **8.16** | **8.21** |

Table B. Asymmetry mean values, Experiment 2

| **Axes** |  | **Humans** | **Robots with high human-likeness** | **Robots with low human-likeness** |
| --- | --- | --- | --- | --- |
| Shoulders |  | 4.01 | 3.29 | 1.76 |
| Elbows |  | 7.76 | 6.89 | 12.46 |
| Hands |  | 26.20 | 18.73 | 22.97 |
| Hips |  | 5.11 | 2.68 | 3.58 |
| Asymmetry Index |  | **10.77** | **7.89** | **10.19** |
